# Evaluation of Two Methods for Decellularizing Human Amniotic Membranes for Use in Tissue Engineering

**DOI:** 10.3390/mps9050127

**Published:** 2026-08-28

**Authors:** Elga Johanna Vargas, Carlos Domínguez-Paz, Lina Andrea Gómez

**Affiliations:** 1 Surgical Pathology Research Group, Facultad de Medicina, Universidad de La Sabana, Chía 250001, Colombia; elga.vargas@unisabana.edu.co; 2 Department of Prototypes and Manufacturing, Grupo de Energía, Materiales y Ambiente (GEMA), Universidad de La Sabana, Chía 140013, Colombia; carlos.dominguez2@unisabana.edu.co; 3 Biomedical Research Center (CIBUS), Facultad de Medicina, Universidad de La Sabana, Chía 250001, Colombia

**Keywords:** human amniotic membrane, decellularization, thermolysin, trypsin-EDTA, extracellular matrix, biomechanical properties

## Abstract

Background: The human amniotic membrane (hAM) is widely used in tissue engineering due to its low immunogenicity and extracellular matrix (ECM) rich in structural proteins; in this context, decellularization aims to remove cellular components while preserving tissue integrity, and this study aimed to compare the effects of two enzymatic methods (trypsin and thermolysin). Methods: Human amniotic membranes obtained from placentas were divided into three groups: non-decellularized control, trypsin-treated, and thermolysin-treated. The membranes were treated with 0.25 trypsin-EDTA for 60 min at 37 °C, followed by incubation with Triton X-100 for 30 min at 37 °C, or with 125 µg/mL thermolysin for 9 min at 37 °C. Histological analysis with hematoxylin–eosin and semi-quantitative scoring assessed epithelial and stromal integrity, immunohistochemistry evaluated laminin α and fibronectin expression with quantitative image analysis, and uniaxial tensile testing measured mechanical properties; no preregistration numbers or animal models were reported. Results: Trypsin achieved more effective epithelial removal, whereas thermolysin caused greater stromal disruption; no statistically significant differences in laminin α or fibronectin expression were found among groups (*p* > 0.05), indicating preservation of key ECM components; both treatments reduced mechanical performance, with thermolysin decreasing early- and mid-path stiffness and trypsin reducing late-path stress and work density. Conclusion: Both enzymatic protocols preserved ECM composition but induced distinct structural and mechanical alterations, suggesting that method selection should balance efficient cell removal with preservation of structural and functional properties according to the intended application.

## 1. Introduction

The human amniotic membrane (hAM) is the innermost layer of the fetal sac and is in contact with the amniotic fluid. It consists of an epithelial layer, a stromal layer, and a basal membrane that separates them. This translucent structure, which lacks blood vessels and nerves, varies in thickness. It is rich in collagen, laminin, and fibronectin, making it valuable in regenerative medicine. After childbirth, the hAM is considered surgical waste, which facilitates its collection [1].

HAM has been used as a biomaterial in surgical reconstruction procedures for nearly a century. In 1910, John Davis first employed it in a skin graft [2]. Its first documented use in ophthalmology occurred in 1940 to treat ocular burns. In 1990, Kim and Tseng popularized its application in ophthalmic practice due to its success in repairing corneal defects [3]. Since then, this natural scaffold has been widely used as a dressing for tissue regeneration and currently has applications in neurosurgery, orthopedics, dermatology, and reconstructive plastic surgery, both in its fresh and decellularized forms [4].

This membrane, used as a biological scaffold, possesses remarkable properties such as antimicrobial capacity, anti-inflammatory effects, low immunological response, and the potential to promote blood vessel formation. When applied as a dressing, hAM reduces scarring and inflammation, promotes epithelialization, minimizes the loss of fluids, proteins, heat, and energy, and contributes to decreased morbidity. These properties, along with their relatively low cost, have positioned them as a promising therapeutic option for temporary coverage [5].

HAM is processed and stored in various ways, and it may contain cells or be decellularized. The removal of cells from this tissue aims to preserve the three-dimensional architecture and biochemical composition of the extracellular matrix (ECM) as much as possible, while eliminating the risk of immunological rejection. This matrix acts as a natural scaffold that guides cell migration, growth, and specialization. It has bactericidal effects and prevents inflammation, making it crucial to maintain its integrity to support an effective regenerative response after application [6].

Epithelium-free hAM promotes cell proliferation and differentiation, structural integrity, and a more uniform pattern of cell growth compared to hAM with intact epithelium [7]. Before clinical application, the hAM donors undergo thorough health screenings, and the amniotic membrane is subjected to a standardized process of collection, processing, and storage [8].

Three-dimensional scaffolds derived from hAM serve as versatile tissue substitutes, usable in both intact and decellularized forms. While intact hAM is commonly applied as a clinical wound dressing, its decellularized counterpart is widely used in tissue engineering due to reduced immunogenicity. The decellularization process aims to eliminate epithelial cells while preserving the basement membrane’s structural and biological integrity. Various protocols—physical, chemical, and biological—have been developed to achieve this, though the optimal method must effectively remove cellular components with minimal disruption to the extracellular matrix [9].

Existing research outlines multiple techniques for preserving, sterilizing, and removing epithelium from hAM, each affecting physical and biological characteristics differently. As a result, defining a universally standardized decellularization protocol remains a complex challenge.

This study evaluates two enzymatic approaches for decellularizing hAM, developing a simple and effective method for their preservation and decellularization, and assessing their performance through various biomechanical and histological analyses for future use as biological dressings.

## 2. Materials and Methods

### 2.1. Obtaining and Transporting Placentas

Thirteen human placentas from healthy mothers were collected at the Hospital Universitario La Samaritana (HUS) in Zipaquirá, Cundinamarca, Colombia, with approval from the ethics committee (record 11-2020). The processing and storage of hAM were carried out at the Research Laboratory of Universidad de La Sabana (Medical Research Center). Donor selection and general conditions for the collection, processing, and storage of hAM followed national guidelines established in Colombia under Resolution 5108 of 2005, which regulates best practices in tissue banks.

Women were excluded if they presented with unregulated pregnancies, any form of maternal or fetal infection (acute or chronic), inflammation or infection of the fetal membranes, recent tattoos or piercings within the past six months, high-risk sexual behavior, physical or mental disabilities, or if the donor was a pregnant woman under the age of 18. The obstetrician verified that the mothers had undergone at least three prenatal check-ups and met the inclusion criteria. Only women who signed the informed consent were included. The study was conducted in accordance with the principles of the Declaration of Helsinki. Placentas were obtained exclusively from mothers who delivered via scheduled cesarean sections, allowing for safe, sterile, and controlled extraction of placental tissue [8].

Inside the operating room, the placenta was transferred into a sterile bag containing 300 mL of chilled sterile saline solution supplemented with antibiotics and antifungals (penicillin 10,000 units/mL, streptomycin 10,000 µg/mL, and amphotericin B 25 µg/mL; Gibco, Thermo Fisher Scientific, Waltham, MA, USA). This initial bag was then sealed within a second sterile bag and subsequently placed into a sterile, airtight plastic container. Both the inner bag and the container were labeled with the donor’s identification details, including name, ID type and number, age, and information regarding the location, date, and time of placenta collection. Finally, the container was refrigerated at a temperature b 2 and 8 °C and processed within a maximum of two hours after the cesarean delivery.

During the cesarean procedure, 5 mL of maternal blood was collected in a tube without an anticoagulant to extract serum, which was then refrigerated alongside the placenta to preserve the cold chain. Upon arrival at the Medical Research Laboratory of the University of La Sabana, the process of dissection of the amniotic membrane commenced. Simultaneously, the blood sample was transferred to the clinical laboratory for testing, including HIV types I and II (Bio-Rad^®^ Laboratories Inc., Hercules, CA, USA), hepatitis A, B, and C (Abbot^®^, Chicago, IL, USA), antibodies against Trypanosoma cruzi, Toxoplasma IgG and IgM (VirClia^®^, Vircell Microbiologist, Granada, Spain), and blood typing (Bio-Rad Laboratories, Inc., Hercules, CA, USA).

### 2.2. Processing of Human Amniotic Membranes

Under biological safety conditions, the placenta bags were opened, and samples of the transport solution were taken for microbiological testing. Cultures for aerobic and anaerobic bacteria, as well as fungi, were performed using various media: 5% sheep blood agar and 5% CO_2_ chocolate agar (incubated for 48 h at 35 °C), thioglycolate broth (5 days at 35 °C), SNVS agar (Schaedler Vitamin K + Sheep Blood) and SCS agar (Schaedler Cysteine + Sheep Blood) under anaerobic conditions (7 days at 35 °C), and Sabouraud agar for fungi (7 days). Results were read every 24 h. All cultures were negative.

The initial processing step involved manually separating the human amniotic membrane (hAM) from the chorionic membrane inside a class II A2 biological safety cabinet. The second step consisted of washing the hAM by gently agitating them in a 0.9% saline solution supplemented with antibiotics and antifungals for 3 min, repeated three times with a freshly prepared solution. Following this, the hAM was placed on a sterile field for the manual removal of sidle debris, blood, and clots. The placenta was then examined macroscopically to exclude anomalies and confirm overall tissue integrity.

In the third step, the hAM was fractionated by spreading them on a surgical tray and cutting them into pieces ranging from 2 × 2 cm to 10 × 10 cm, depending on membrane quality and intended application. Each fragment was subsequently mounted on nitrocellulose paper (Sartorius^®^, Göttingen, Germany), with the epithelial side facing upward and the stromal side in contact with the support.

### 2.3. Storage of Human Amniotic Membranes

As we described previously [8], the hAM, once placed on nitrocellulose paper, was rolled and stored in Falcon^®^ tubes containing a freezing solution of 50% DMEM (Dulbecco’s Modified Eagle Medium) Low Glucose and 50% glycerol (Sigma-Aldrich, St. Louis, MO, USA). Samples of the solutions and tissues were taken for microbiological and histological analysis. Cultures for aerobic and anaerobic bacteria were performed using various agars and incubation methods, as we described previously. The hAM was stored at −20 °C until mechanical and histopathological testing. Before analysis, the membranes were thawed at 4 °C for 6 h, fixed in formalin for 24 h, and transported in a cooler to the histological testing lab at Universidad de La Sabana.

### 2.4. Decellularization of Human Amniotic Membranes

#### 2.4.1. Trypsin-Based Decellularization

To decellularize hAMs using the enzyme trypsin, the membranes were first removed from the freezer (−20 °C), where they had been stored in a 1:1 mixture of DMEM and glycerol. They were then washed with sterile saline solution (1X PBS) five times. Subsequently, the membranes were incubated in a 0.25% trypsin-EDTA solution (Merck, Darmstadt, Germany) at 37 °C for 60 min under gentle constant agitation. The enzymatic reaction was halted by adding DMEM supplemented with 10% fetal bovine serum. Triton X-100 (Thermo Fisher Scientific, Waltham, MA, USA) was then added to the hAMs, followed by incubation with agitation for 30 min at 37 °C. The membranes were washed five times with PBS and stored at 4 °C.

#### 2.4.2. Thermolysin-Based Decellularization Within the Range Commonly Used for Amniotic and Fetal Membrane Testing

The membranes were removed from the freezer (−20 °C), where they had been stored in a 1:1 mixture of DMEM and glycerol and rinsed several times with sterile saline solution (1X PBS). Subsequently, the hAM samples were incubated in a 125 µg/mL thermolysin solution (Sigma Chemicals, St. Louis, MO, USA) at 37 °C for 9 min under gentle agitation. The enzymatic reaction was halted using DMEM supplemented with 10% fetal bovine serum. The membranes were washed five times with 1X PBS, then the hAM samples were stored at 4 °C.

### 2.5. Histological Characterization of Decellularized Human Amniotic Membranes

#### 2.5.1. Conventional Evaluation

Four decellularized human amniotic membranes (dhAMs) prepared using trypsin, four dhAMs prepared using thermolysin, and two non-decellularized human amniotic membranes (nhAM) were placed in properly labeled vials containing 10 mL of 10% buffered formaldehyde. The samples were fixed for 24 h, processed using conventional techniques, and histological slides were obtained. These were stained with hematoxylin and eosin (H&E) for analysis.

The AxioScope A1 optical microscope (Carl Zeiss MicroImaging GmbH, Göttingen, Germany). was used to observe the slides, which evaluate the decellularization process. Three foci were selected at 40× magnification, and images were acquired using a Carl Zeiss ICc5 camera (Carl Zeiss Microscopy GmbH, Göttingen, Germany) and analyzed using ZEN 2.3 software (Carl Zeiss Microscopy GmbH, Jena, Germany) connected to the microscope. Then, the integrity score for epithelim and stroma described by M. Wagner et al. [10] was applied for each focus. The integrity score classifies the epithelium based on its continuity and the clear differentiation of the basement membrane into 4 categories with a score from 0 to 3, 0 being normal histology and 3 being tissue that is impossible to evaluate given the complete loss of tissue differentiation. Similarly, the integrity of the stroma is classified into 4 categories with a score from 0 to 3, with 0 being a stroma composed of continuous, lamellar collagen fibers and 3 being the complete disruption of the tissue. Because the scoring system generated ordinal data and the number of samples per group was small, comparisons among the three groups were performed using the non-parametric Kruskal–Wallis test. When statistically significant differences were detected, post hoc pairwise comparisons were conducted using the Wilcoxon rank-sum test with Bonferroni correction for multiple comparisons. Statistical significance was set at *p* < 0.05.

#### 2.5.2. Immunohistochemical Evaluation

To evaluate the impact of decellularization on the composition of the basal membrane of hAMs, immunohistochemistry was performed using Laminin alpha (monoclonal mouse antibody IgG1 Clone # 546215, R&D Systems) and Fibronectin (monoclonal mouse antibody, clone 568, Novus Biologicals) both diluted 1:100. Two dhAM samples—one for each enzyme—and one nhAM sample, used as a control, were analyzed.

Sections of 5 μm thickness were obtained from paraffin-embedded samples using a LEICA^®^ Biosystems microtome and mounted on slides previously treated with an albumin–glycerol mixture. After deparaffinization and rehydration, the sections were subjected to pretreatment with hyaluronidase. The samples were incubated overnight at 4 °C with primary antibodies, and the endogenous peroxidase activity was blocked with 0.5% hydrogen peroxide. The sections were incubated with the secondary antibody for 45 min at room temperature. The antigen–antibody complexes were visualized with diaminobenzidine (DAB), and the cells were lightly stained with Mayer’s hematoxylin (MH). Finally, the sections were mounted with an aqueous medium for microscopic observation. The AxioScope A1 optical microscope (LEIKA^®^) was used to observe each slide, and three non-overlapping high-power fields (HPFs) were randomly selected from representative areas of the tissue section to account for intrasection heterogeneity, avoiding necrotic, artefactual, or edge regions. Images of these three points were captured using a Carl Zeiss^®^ IC C5 camera and ZEN^®^ software connected to the microscope, and images were saved and analyzed in uncompressed TIFF format. Quantitative image analysis was performed using Fiji/ImageJ software (version 2.14.0/1.54f, National Institutes of Health, Bethesda, MD, USA).

A uniform processing workflow, including color deconvolution to separate DAB signal from hematoxylin background, and a uniform intensity threshold, was applied to all images within each experimental set using the “Image→Adjust→Threshold” function to ensure comparability between groups. After thresholding, images were converted to binary format using “Process→Binary→Make Binary”. Quantitative measurements included positive area, area fraction (%), and integrated optical density (IOD). IOD was calculated as the product of the positive area and the mean gray value. IOD values were obtained from three representative microscopic fields for each condition. Data were expressed as mean values.

### 2.6. Mechanical Evaluation of Decellularized hAM

#### Tensile Testing and Behavior-Aligned Mechanical Analysis

Tensile tests generated force–displacement curves that were converted to engineering stress–strain, with stress computed from force and cross-sectional area and strain from change in length over the gauge length, following standard soft-tissue testing practice [11,12]. Small preload offsets were removed by baseline correction, and the signals were lightly smoothed with a symmetric Savitzky–Golay filter to stabilize derivatives while preserving the initial slope [13]. Uniaxial tensile tests were performed using a universal testing machine (Instron 3900, Instron Ltd., High Wycombe, UK) equipped with a 5 kN load cell and operated at a constant crosshead speed of 3 mm/min, within the range commonly used for amniotic and fetal membrane testing [11,14,15].

For comparability across specimens and treatments, only the monotone loading branch from the onset of effective loading to ultimate tensile stress (UTS) was analyzed. Onset was defined as the point where stress rose above noise, and the stress–strain curve showed a consistently positive slope. Each curve was then re-referenced so that onset corresponded to ((\varepsilon’, \sigma’) = (0, 0)), cropped to the onset→UTS segment, and reparametrized on a normalized strain coordinate (\xi\in [0, 1]). From these aligned curves, tangent and secant moduli at selected points, point stresses along the loading path, work density up to UTS, and the strain span from onset to UTS were extracted.

Statistical analyses were performed on the mechanical metrics extracted from the behavior-aligned stress–strain curves using Python (Python 3.10.19). Given the limited number of specimens per experimental group, no assumption of normality was made, and non-parametric inference was adopted throughout the mechanical analysis. Treatment groups were compared with the untreated control using two-sided permutation tests. Effect sizes were quantified using Hedges’ *g*, and 95% bootstrap confidence intervals were estimated to assess the magnitude and uncertainty of the observed differences [16]. Mechanical data are reported as mean ± standard deviation (SD), while *p*-values and effect sizes are presented together to facilitate interpretation of both statistical significance and practical relevance.

## 3. Results

### 3.1. Histological Characterization of Decellularized hAM

The results of the histological integrity assessment of nhAM and dhAM in H&E-stained sections are presented in Table 1. Descriptive statistics calculated using the mean score obtained for each hAM sample are shown in Table 2.

The median epithelial integrity score was 0.17 (Q1–Q3: 0.08–0.25) in the untreated nhAM group, 3.00 (3.00–3.00) in the trypsin-treated dhAM group, and 2.84 (2.67–3.00) in the thermolysin-treated dhAM group. A statistically significant overall difference was detected among the groups for the epithelial score (Kruskal–Wallis test, H = 6.70, *p* = 0.035). However, none of the pairwise comparisons remained statistically significant after Bonferroni correction.

The median stromal integrity score was 0.00 (0.00–0.00) in the untreated nhAM group, 0.50 (0.25–0.75) in the trypsin-treated group, and 1.00 (0.83–1.00) in the thermolysin treated group. The overall difference in stromal scores was not statistically significant (Kruskal–Wallis test, H = 4.89, *p* = 0.087).

Descriptively, trypsin treatment produced the greatest epithelial disruption, whereas thermolysin treatment showed the highest stromal integrity-loss scores. Nevertheless, statistically significant pairwise differences could not be demonstrated with the available sample size.

The results of quantitative image analysis for fibronectin and laminin α immunohistochemical evaluation are shown in Table 3. IOD analysis of immunohistochemical staining showed no statistically significant differences in fibronectin or laminin expression between treated dhAM samples and the control dhAM sample. For fibronectin, the mean IOD values were 34,961,655 ± 3,117,906 in untreated nhAM, 36,949,768 ± 299,621 in trypsin-treated dhAM, and 35,063,786 ± 2,885,360 in thermolysin-treated dhAM, respectively, with no significant differences compared with control nhAM (*p* = 0.384 and *p* = 0.969, respectively). For laminin α, the mean IOD values were 37,352,305 ± 342,077 in control nhAM, 37,321,284 ± 156,685 in trypsin-treated dhAM, and 37,456,305 ± 96,287 in thermolysin-treated dhAM, with no significant differences compared with control nhAM (*p* = 0.896 and *p* = 0.656, respectively).

**Figure 1:**
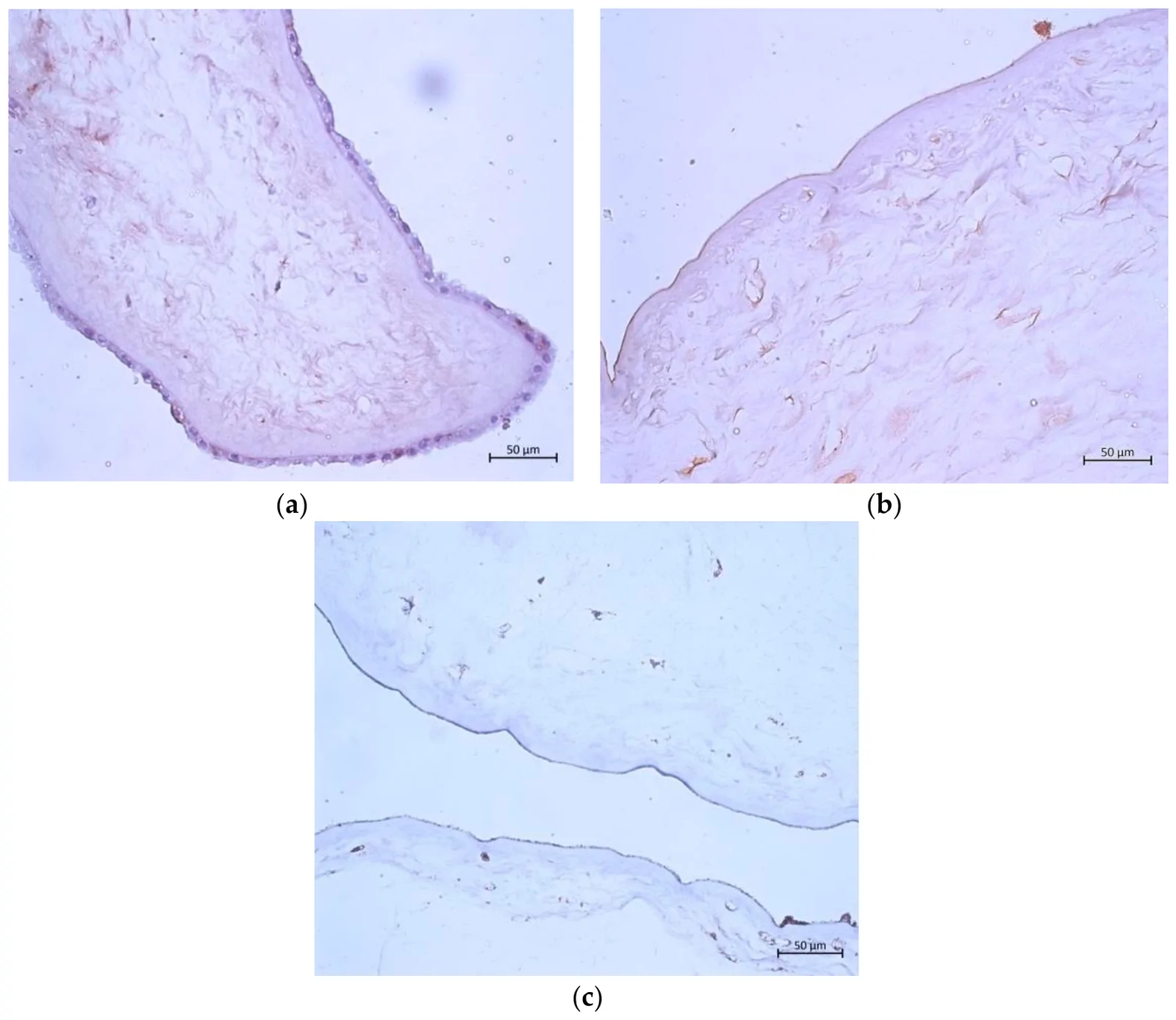
Fibronectin immunolabeling remains detectable after trypsin- and thermolysin-mediated decellularization of human amniotic membrane. (**a**). Non-decellularized hAM (control), DAB & MH, 40×. (**b**). Decellularized hAM with trypsin, DAB & MH, 40×. (**c**). Decellularized hAM with thermolysin, DAB & MH, 40×.

**Figure 2:**
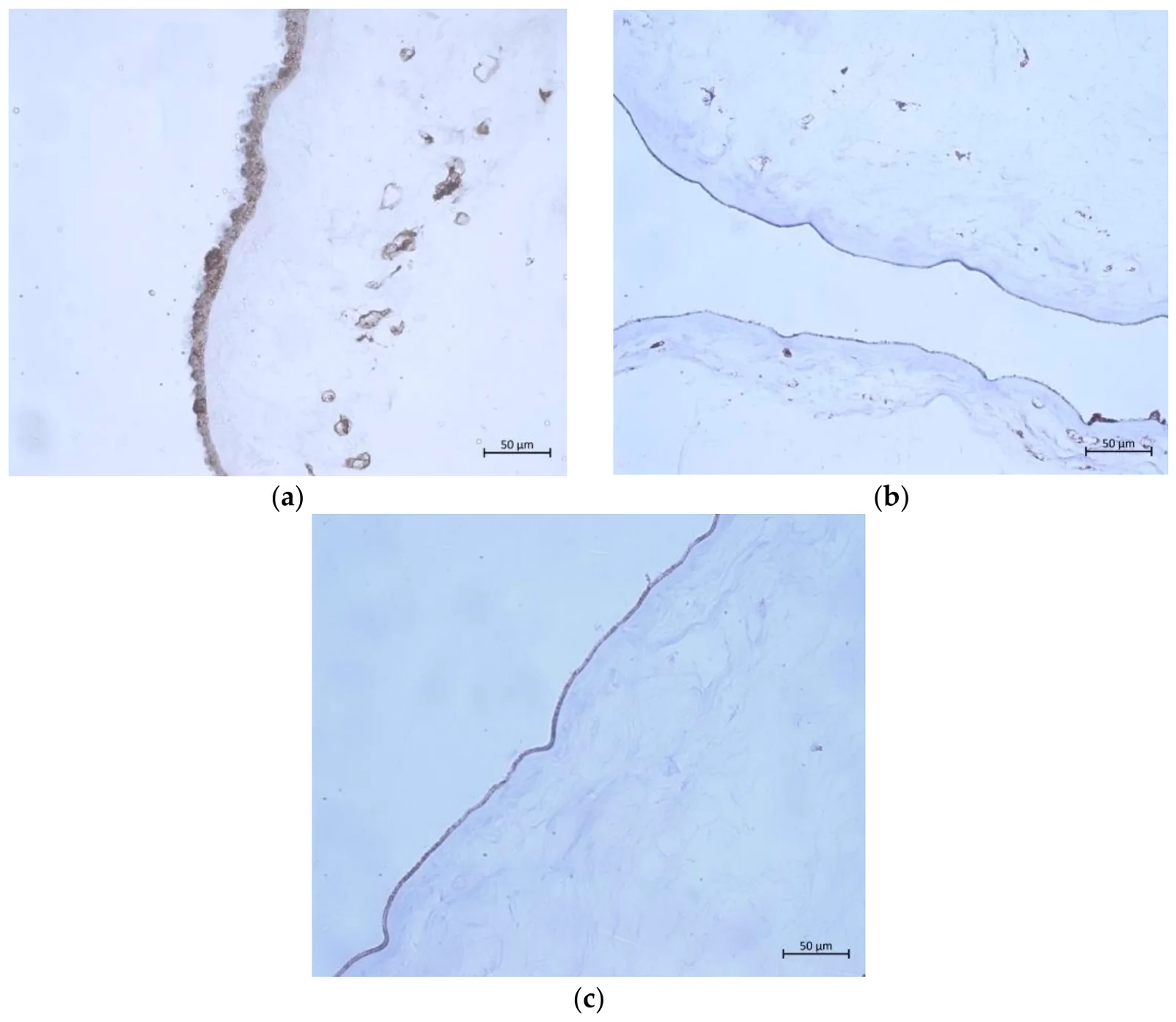
Laminin-α immunolabeling remains detectable after trypsin- and thermolysin-mediated decellularization of human amniotic membrane. (**a**) Non-decellularized. hAM (control), DAB & MH, 40×. (**b**) Decellularized hAM with trypsin, DAB & MH, 40×. (**c**) Decellularized hAM with thermolysin, DAB & MH, 40×.

The field-level measurements showed similar immunolabeling values among the evaluated conditions, a finding that is also visually evident in the representative images shown in Figure 1 and Figure 2. However, because the three microscopic fields represented technical subsamples from a single hAM sample per condition, no inferential statistical comparisons were performed. Therefore, these observations should be interpreted de-scriptively and do not demonstrate statistical equivalence among the treatments.

### 3.2. Mechanical Response of Decellularized hAM

After behavior-aligned normalization from the onset of effective loading to ultimate tensile stress (UTS), the mechanical response differed among groups. The nonlinear, J-shaped stress–strain behavior observed here is consistent with tensile tests on human amniotic membrane, which describe low initial stiffness and progressive stiffening up to failure [1,15,17]. Relative to hAM + Untreated, both decellularization protocols reduced the overall response, although with distinct patterns. Similar treatment-related reductions have been described for decellularized hAM scaffolds processed with enzymatic or detergent-based protocols [7,11,18,19]. hAM + Thermolysin showed the clearest reduction in early- and mid-path stiffness, whereas hAM + Trypsin exhibited the lowest late path stress and work density. Group-wise mean ± SD values for the principal scalar metrics are summarized in Table 4, treatment versus control comparisons is presented in Table 5, and the onset to UTS strain span is summarized in Table 6.

In addition to the behavior-aligned metrics, conventional tensile outcomes were consistent with published ranges for human amniotic membranes. Ultimate tensile stress and strain at failure for hAM + Untreated (non-decellularized) lay within the lower part of the 1–8 MPa and sub-0.4 strain ranges reported for native hAM, reflecting the use of hydrated strips and ex vivo storage [15,17,20]. Both decellularized groups showed modest reductions in UTS and ε fail relative to the untreated condition, similar in magnitude to changes described for enzymatically or detergent-treated hAM scaffolds and amniotic-derived grafts [7,11,18,19].

### 3.3. Behavior-Aligned Mean Curves (Onset→UTS)

**Figure 3:**
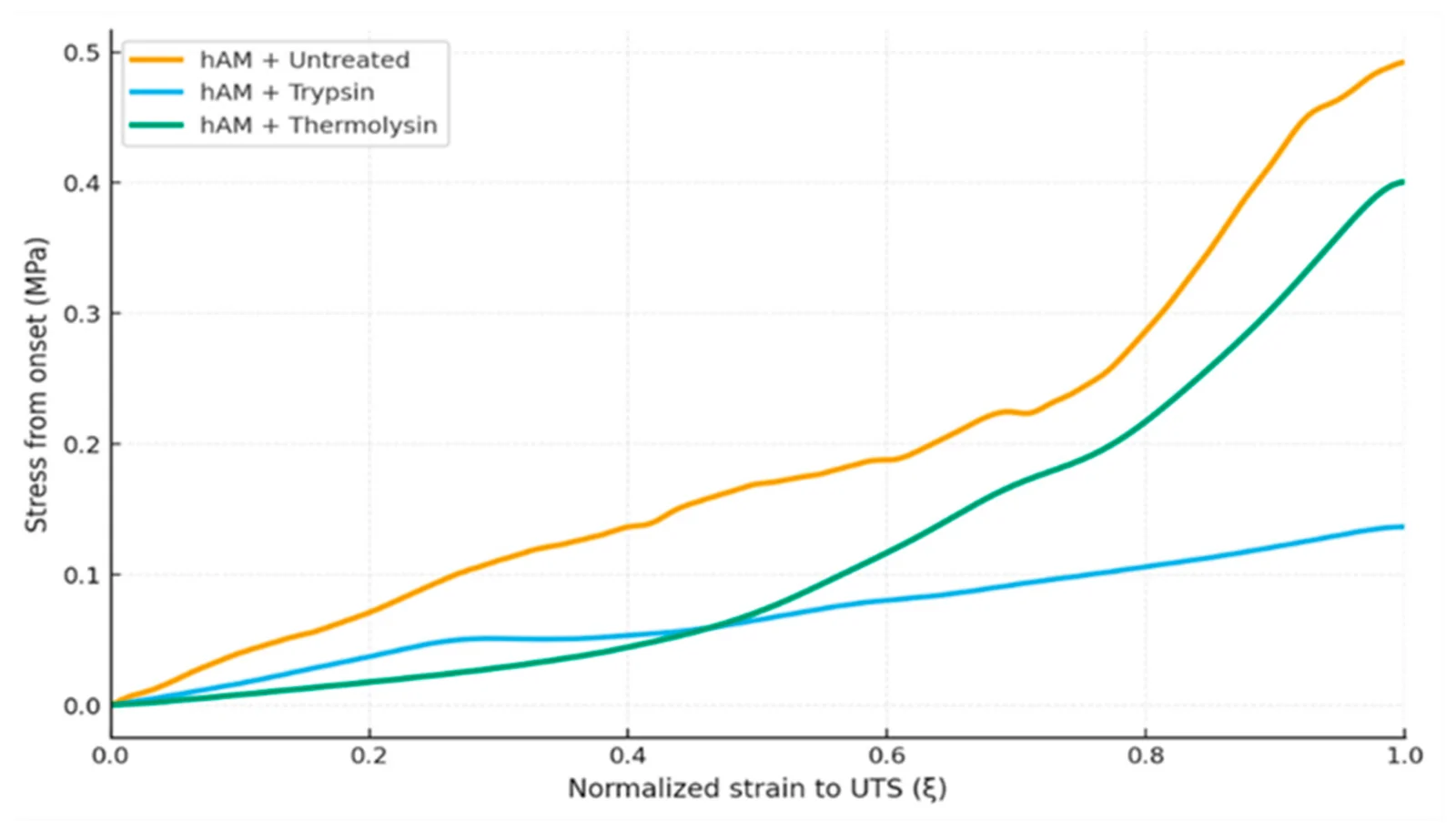
Group mean curves aligned by behavior (onset→UTS).

All curves were aligned at the onset of effective loading (ε′ = 0, σ′ = 0) and truncated at UTS (ξ = 1). This normalization reduced toe-region variability and enabled direct comparison of the monotone loading branch across groups. Similar alignment approaches have been reported in biomechanical analyses of fetal membranes to account for pre-strain and toe-region variability. The mean curves showed that hAM + Untreated maintained the highest resistance growth throughout the loading path, hAM + Thermolysin followed an intermediate trajectory with a late increase, and hAM + Trypsin remained the lowest throughout most of the normalized loading path (Figure 3).

**Figure 4:**
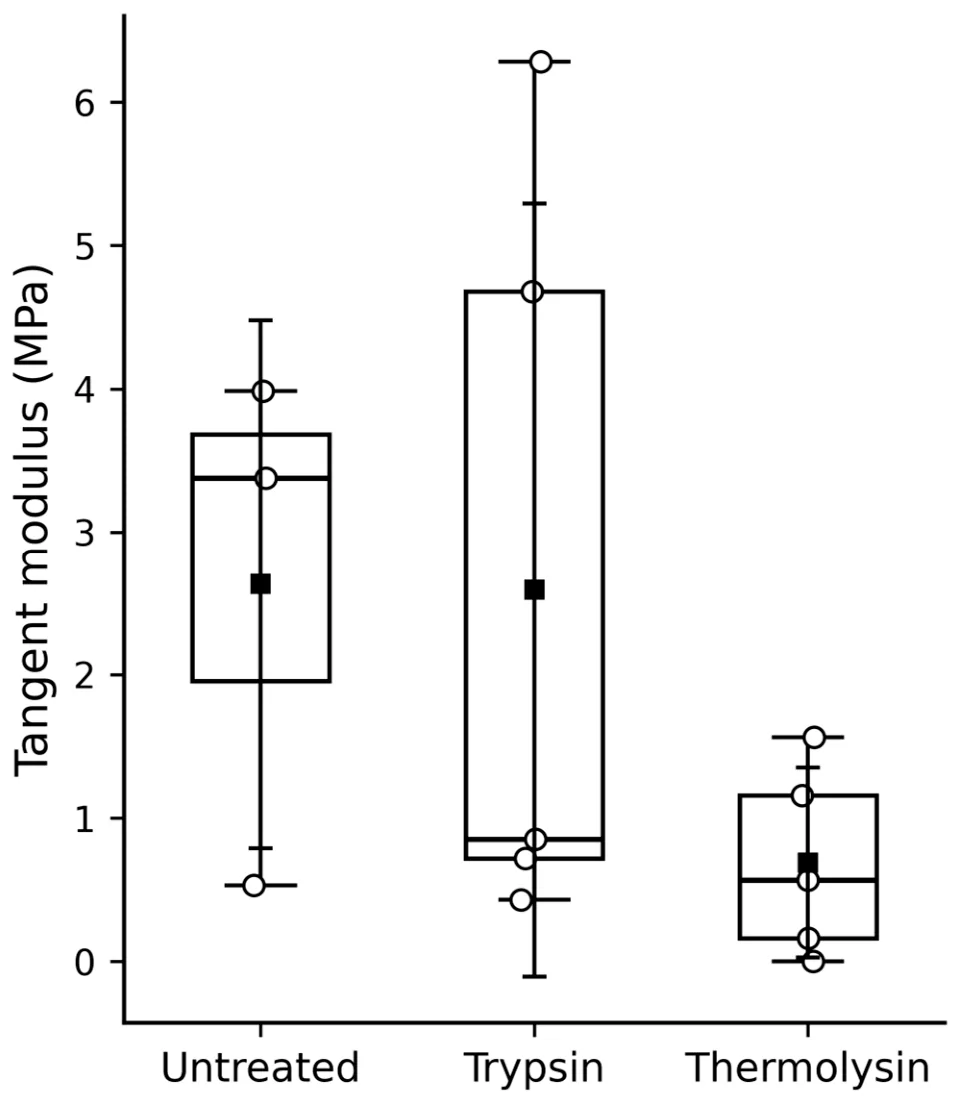
Tangent modulus measured at ξ = 0.05 for untreated hAM, hAM treated with trypsin, and hAM treated with thermolysin. Boxplots summarize the distribution of the measurements, individual points represent independent specimens, and statistical annotations correspond to comparisons against the untreated control.

For early local stiffness, quantified by the tangent modulus at ξ = 0.05, mean values were 2.63 ± 1.84 MPa for hAM + Untreated, 2.59 ± 2.70 MPa for hAM + Trypsin, and 0.69 ± 0.66 MPa for hAM + Thermolysin (Table 4; Figure 4). These magnitudes fall within the lower range of tensile moduli reported for native hAM in uniaxial tests, which typically span from approximately 1 to 8 MPa depending on region, orientation, and hydration [17,20,21,22]. Relative to hAM + Untreated, hAM + Trypsin showed no measurable difference (Δ ≈ −0.04 MPa, *p* = 0.967, Hedges’ g = −0.01), whereas hAM + Thermolysin showed a marked reduction (Δ ≈ −1.94 MPa, *p* = 0.070, g = −1.41).

**Figure 5:**
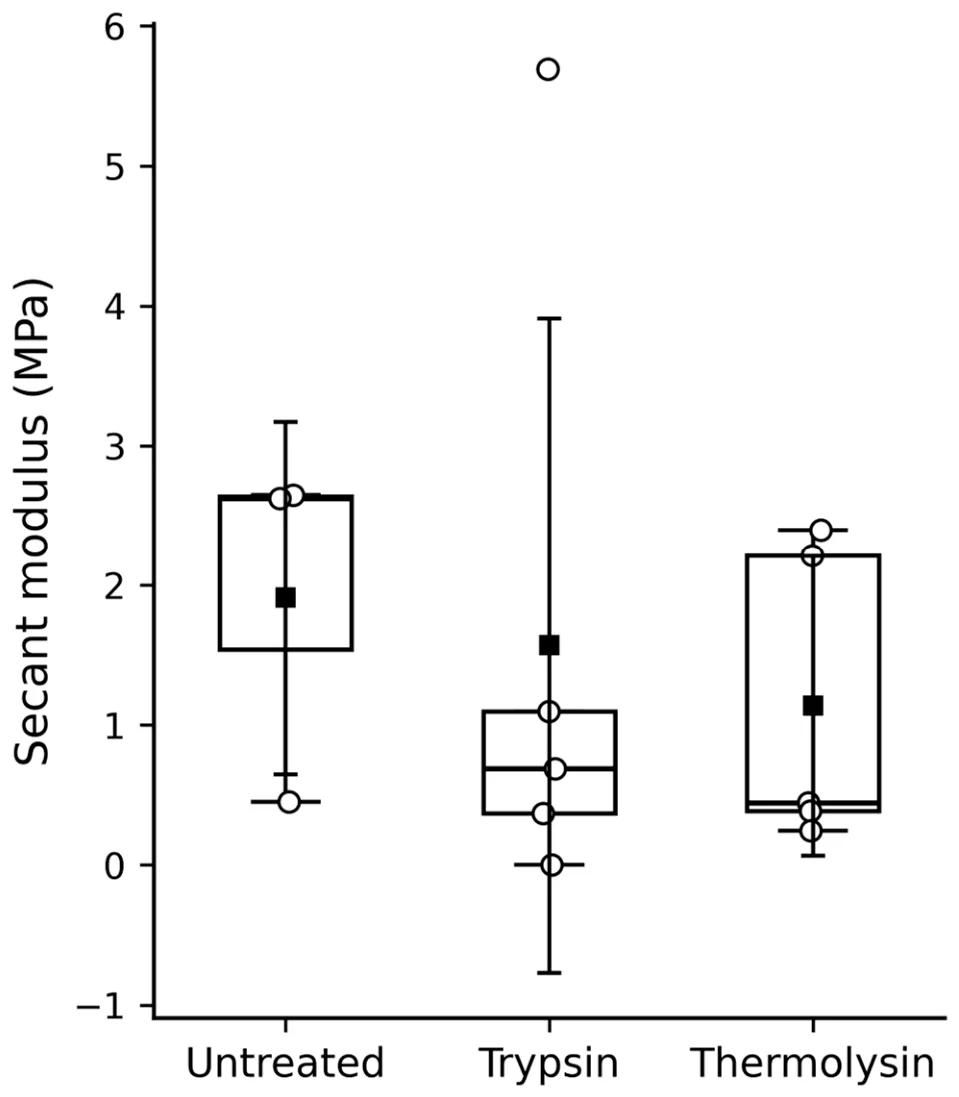
Secant modulus measured at ξ = 0.50 for untreated hAM, hAM treated with trypsin, and hAM treated with thermolysin. Boxplots summarize the distribution of the measurements, and individual points represent independent specimens.

For mid-path effective stiffness, represented by the secant modulus at ξ = 0.50, the corresponding means were 1.91 ± 1.26 MPa, 1.57 ± 2.34 MPa, and 1.14 ± 1.07 MPa for hAM + Untreated, hAM + Trypsin, and hAM + Thermolysin, respectively (Table 4; Figure 5). These values are comparable to mid-range effective moduli reported for hydrated hAM and amnion-derived grafts used clinically [15,22]. The reduction was negligible for hAM + Trypsin (Δ ≈ −0.34 MPa, *p* = 0.966, g = −0.14) and moderate for hAM + Thermolysin (Δ ≈ −0.77 MPa, *p* = 0.165, g = −0.59).

**Figure 6:**
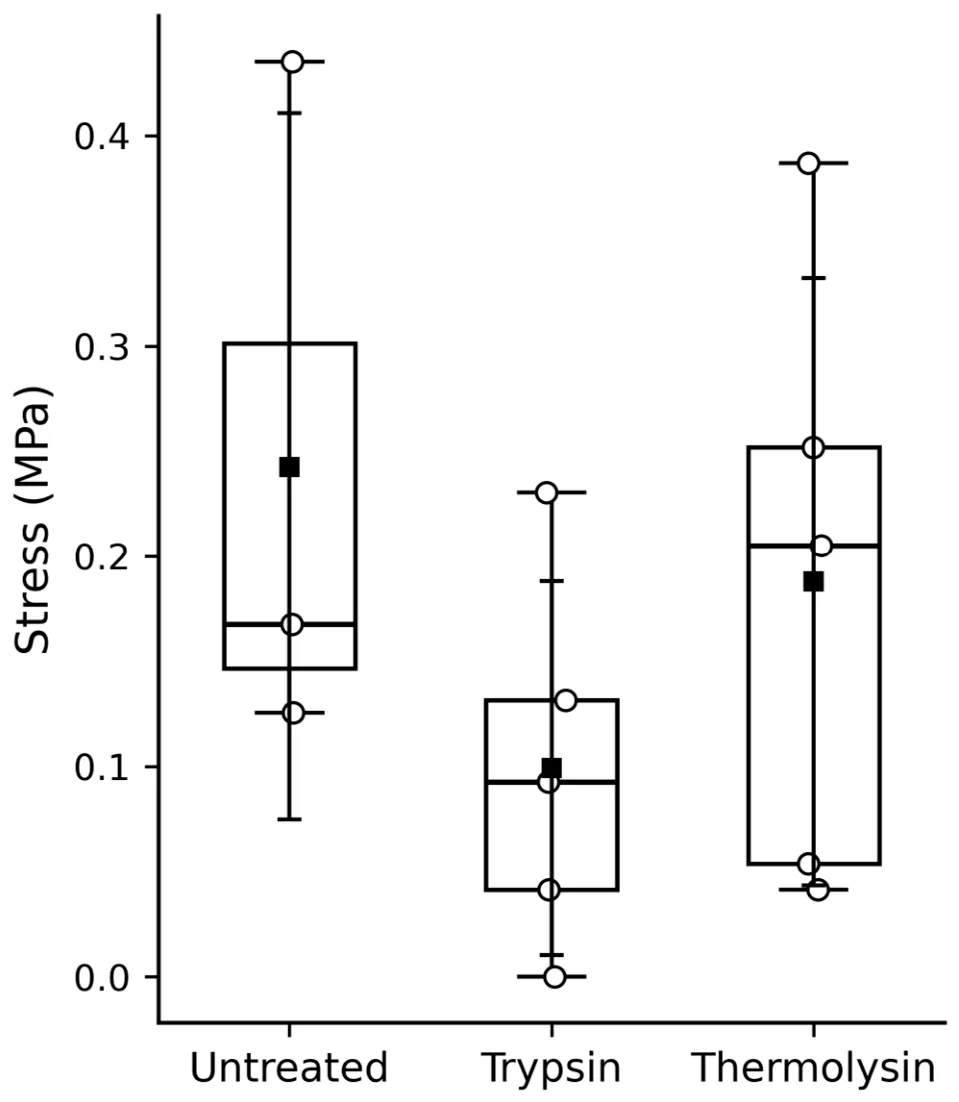
Stress measured at ξ = 0.75 for untreated hAM, hAM treated with trypsin, and hAM treated with thermolysin. Boxplots summarize the distribution of the measurements, individual points represent independent specimens, and statistical annotations correspond to comparisons against the untreated control.

**Figure 7:**
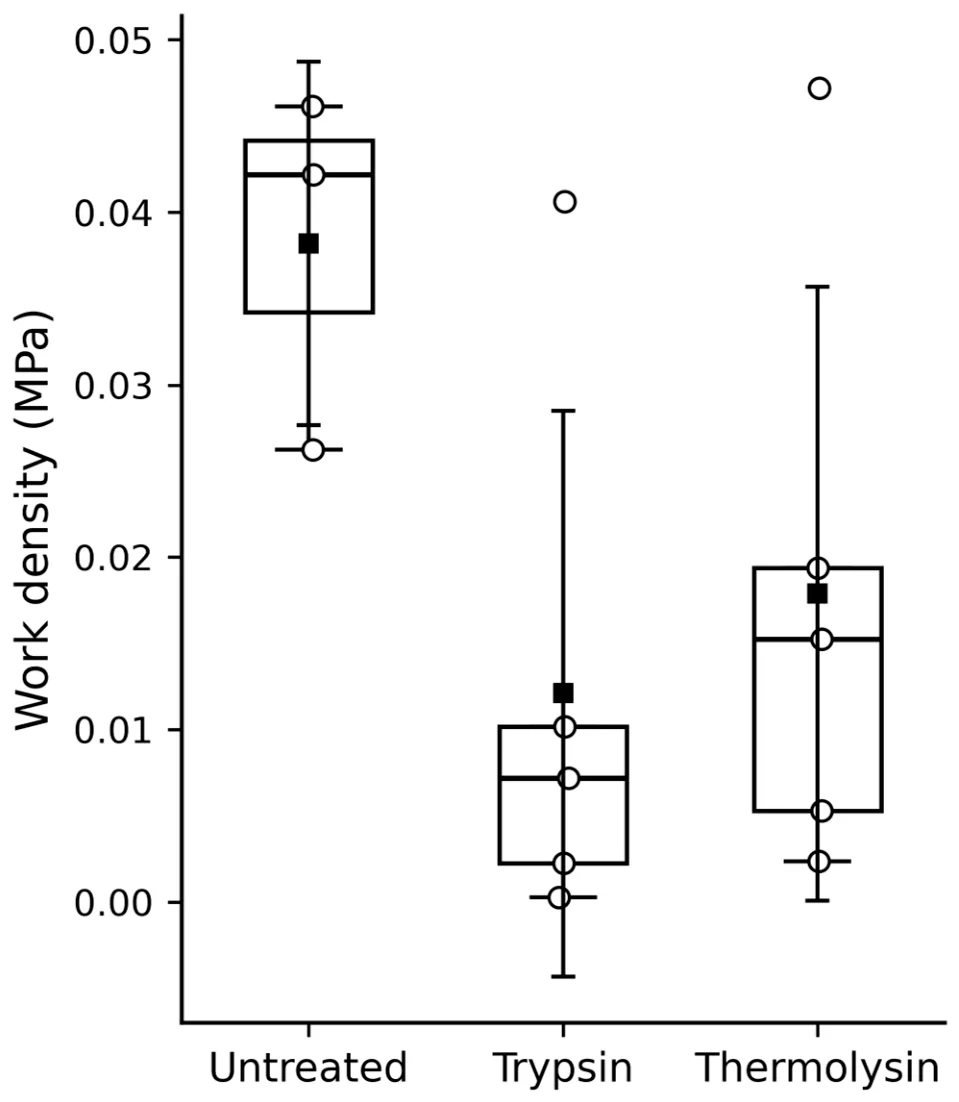
Work density from loading onset to ultimate tensile stress (UTS) for untreated hAM, hAM treated with trypsin, and hAM treated with thermolysin. Boxplots summarize the distribution of the measurements, individual points represent independent specimens, and statistical annotations correspond to comparisons against the untreated control.

Late-path resistance and energy absorption showed a different pattern. Stress at ξ = 0.75 was lowest in hAM + Trypsin (0.099 ± 0.089 MPa) compared with hAM + Untreated (0.243 ± 0.168 MPa), corresponding to a large negative effect size (Δ ≈ −0.14 MPa, *p* = 0.160, g = −1.03), whereas hAM + Thermolysin showed a smaller reduction (0.188 ± 0.144 MPa; Δ ≈ −0.055 MPa, *p* = 0.646, g = −0.31) (Figure 6). Work density was also reduced in both treated groups relative to hAM + Untreated (0.0382 ± 0.0105 MPa), with means of 0.0121 ± 0.0164 MPa for hAM + Trypsin and 0.0179 ± 0.0178 MPa for hAM + Thermolysin (Figure 7). Comparable reductions in energy absorption after decellularization have been reported when comparing native and processed hAM or amnion-derived grafts [18,21]. These changes corresponded to large negative effect sizes for both hAM + Trypsin (Δ ≈ −0.026 MPa, *p* = 0.073, g = −1.54) and hAM + Thermolysin (Δ ≈ −0.020 MPa, *p* = 0.139, g = −1.12).

Overall, although most permutation tests did not cross the conventional α = 0.05 threshold, the effect-size estimates consistently indicated treatment-related softening, with more pronounced early stiffness loss in hAM + Thermolysin and greater late-path and energy loss in hAM + Trypsin. These trends are visually evident in the aligned mean curves and the distributions shown in Figure 3, Figure 4, Figure 5, Figure 6 and Figure 7 and are quantified in Table 4, Table 5 and Table 6. Similar moderate-to-large effect sizes without formal statistical significance have been described in small-sample biomechanical studies of fetal membranes [11].

## 4. Discussion

The decellularization of hAM is a critical step that enables the removal of immunogenic elements such as cellular components, membrane antigens, and soluble proteins, thereby improving its biocompatibility and supporting its use as a natural scaffold or wound dressing in regenerative medicine [5,19].

Decellularization can be performed using physical, chemical, and enzymatic methods, each with its own advantages and limitations, in terms of efficiency and preservation of the ECM. The enzymatic method has proven particularly effective for the selective removal of cellular components, largely maintaining the structural and biochemical integrity of the tissue [23,24].

The results of the present study demonstrate that the decellularization methods evaluated induce distinct patterns of tissue damage, differentially affecting epithelial and stromal compartments. Descriptively, trypsin treatment produced the greatest epithelial disruption, whereas thermolysin treatment showed the highest stromal integrity-loss scores. This finding is consistent with the known mechanism of action of trypsin, a serine protease that cleaves peptide bonds in a relatively nonspecific manner, particularly targeting cell adhesion proteins and facilitating epithelial detachment [6,25]. On the contrary, its action appears to be less aggressive on the ECM, which would explain the moderate stromal damage observed.

In contrast, thermolysin exhibited an inverse pattern, with greater stromal disruption (median 1, IQR 0, 83–1) and relatively intermediate epithelial damage (median 2, 84, IQR 2.67–3). Thermolysin is a metalloproteinase with specificity for hydrophobic amino acid residues, and it has been shown to preferentially degrade structural components of the ECM, including collagen-associated proteins [16,26]. This enzymatic specificity may account for the pronounced stromal alterations observed, such as fiber fragmentation and edema.

These findings suggest that the choice of decellularization agent should be guided by the intended application of the processed tissue. Trypsin may be more suitable when preservation of stromal architecture is desired. Conversely, thermolysin can be advantageous in applications that require stromal remodeling or removal while partially preserving epithelial structures.

Our findings of the immunohistochemical assessment are consistent with previous reports showing that enzymatic denudation of hAM can preserve basement membrane components, particularly laminin, depending on the protocol used [19,27,28]. Trypsin/EDTA has been reported to generate dhAM with preserved basement membrane integrity, including laminin α5 immunodetection [29], whereas thermolysin-based protocols have been shown to preserve basement membrane structural and molecular integrity, including laminin 5 staining [28]. In addition, acellular amniotic membrane matrices have been reported to retain key extracellular matrix proteins, including laminin and fibronectin [30]. The present study evaluated two enzymatic decellularization protocols for hAM, demonstrating that both trypsin and thermolysin effectively preserve ECM composition while producing distinct patterns of structural and mechanical alteration. These findings highlight the importance of selecting a decellularization strategy based on the intended application of biomaterial.

Histological analysis revealed that trypsin achieved a higher degree of epithelial removal compared to thermolysin, confirming its effectiveness as a decellularizing agent. This result is consistent with the known proteolytic activity of trypsin, which targets cell adhesion proteins and facilitates epithelial detachment. In contrast, thermolysin-treated samples showed less epithelial disruption but significantly greater stromal damage, characterized by fiber disorganization and increased integrity scores. These differences were statistically significant and indicate that the enzymatic specificity of each agent plays a critical role in determining the pattern of tissue modification. The inverse relationship observed between epithelial removal and stromal preservation suggests a trade-off between decellularization efficiency and structural integrity. While complete epithelial removal is desirable to reduce immunogenicity, excessive disruption of the stromal matrix may compromise the scaffold’s biomechanical stability and biological performance. In this context, trypsin appears to provide a more favorable balance when preservation of stromal architecture is required, whereas thermolysin may be advantageous in applications that require greater remodeling or partial degradation of the ECM.

Despite the structural differences observed histologically, immunohistochemical analysis demonstrated that the enzymatic protocol significantly altered the expression of key basement membrane proteins, including laminin α and fibronectin. The absence of statistically significant differences in IOD values across groups indicates that both treatments preserved essential ECM components. These findings are particularly relevant, as laminin and fibronectin play crucial roles in cell adhesion, migration, and differentiation. Their preservation supports the potential use of decellularized hAM as a biologically active scaffold in regenerative medicine.

The mechanical evaluation further demonstrated that decellularization affects the functional behavior of hAM, even when ECM composition is preserved. Both treatments led to reductions in mechanical performance compared to native tissue, although the patterns differed between methods. Thermolysin-treated membranes exhibited a marked reduction in early- and mid-path stiffness, suggesting a weakened initial load-bearing capacity. In contrast, trypsin-treated samples maintained early stiffness more effectively but showed reduced stress at later stages of deformation and lower energy absorption capacity.

These differences in mechanical behavior can be interpreted in light of the histological findings. The greater stromal disruption seen with thermolysin likely contributes to the reduction in stiffness, as the collagen network is essential for resisting deformation under load. Conversely, the relative preservation of stromal structure in trypsin-treated membranes may explain the retention of early stiffness, although alterations in matrix organization or crosslinking could account for the reduced performance at higher strains. The consistent trend toward mechanical softening in both treatments, supported by moderate-to-large effect sizes, indicates that decellularization inherently modifies tissue mechanics.

From a translational perspective, these mechanical changes may influence the clinical handling and performance of hAM-based scaffolds. Reduced stiffness may improve flexibility and conformability, which can be advantageous for applications such as wound coverage. However, decreased tensile strength and energy absorption could increase the risk of tearing or failure during surgical manipulation. Therefore, the mechanical profile of the decellularized membrane should be considered alongside histological and biochemical outcomes when selecting a processing protocol.

In summary, the results of this study demonstrate that trypsin and thermolysin produce distinct structural and mechanical outcomes while preserving ECM composition. These findings reinforce the concept that no single decellularization method is universally optimal, and that protocol selection should be guided by the specific requirements of the intended tissue engineering application, balancing efficient cell removal with preservation of structural integrity and functional properties.

### 4.1. Limitations and Future Work

Several limitations of this study should be acknowledged. First, the small sample size, particularly in the control group (n = 3), limits the statistical power and generalizability of the findings. Second, the use of ordinal scoring systems constrains the analytical approaches and may reduce sensitivity in detecting subtle differences. Despite these limitations, the consistency of the observed patterns, reflected in narrow interquartile ranges within groups, supports the presence of a reproducible biological effect associated with each treatment.

Additionally, the use of semi-quantitative histological scoring introduces a degree of subjectivity, and more advanced quantitative imaging techniques could provide greater resolution. Furthermore, although uniaxial tensile testing provides valuable insights into mechanical behavior, additional analyses such as viscoelastic characterization and fatigue testing would be beneficial to fully understand the functional performance of the scaffold.

Future studies should aim to increase sample size, incorporate advanced imaging and molecular analyses, and explore the biological performance of decellularized hAM in vitro and in vivo. Evaluating cell reseeding, tissue integration, and long-term remodeling will be essential to determine the clinical relevance of the observed differences between protocols.

### 4.2. Mechanical Implications

Mechanical analysis indicated that the two decellularization methods affected hAM differently. hAM + Thermolysin showed the clearest reduction in early- and mid-path stiffness, suggesting a greater effect on the initial load-bearing response of the membrane. In contrast, hAM + Trypsin preserved early stiffness more closely to the untreated condition but exhibited lower late-path stress and reduced work density. Previous studies on decellularized hAM have similarly shown that enzymatic and detergent-based protocols can differentially affect stiffness- and failure-related properties [7,11]. From a translational perspective, these patterns may be relevant for handling, drape, and resistance to tearing during surgical manipulation. Reduced early tangent stiffness may alter the way the membrane engages under small deformations, whereas lower late-path stress and work density suggest a reduced capacity to sustain and absorb load before failure. Although most comparisons did not reach statistical significance, likely due to the limited sample size and the inherent biological variability of hAM, several effect sizes were moderate to large and consistently negative, supporting the interpretation that both treatments altered membrane mechanics, albeit in different ways.

#### 4.2.1. Extensibility from Onset to UTS

The onset-to-UTS strain span (ϵ’UTS) served as an index of the useful extensibility of each group. Mean values were 0.305 ± 0.078 for hAM + Untreated, 0.258 ± 0.195 for hAM + Trypsin, and 0.193 ± 0.056 for hAM + Thermolysin, indicating the shortest useful deformation range in the Thermolysin group. These values are broadly consistent with reported failure strains for human amniotic membrane in planar tensile and fracture-related mechanical tests, which generally remain below 0.4 in engineering strain [17,20,21].

#### 4.2.2. Practical Implications

Changes in early tangent modulus may influence handling and suture pull-through risk, since a higher (E_{tan}) near onset suggests faster load transfer to the matrix/fiber network. In this context, differences in early stiffness may affect how readily the membrane engages under small deformations during placement and fixation. Likewise, differences in mid- and late-path resistance, together with work density, indicate how each treatment may alter the balance between stiffness and deformability. These features are relevant for graft drape, tear onset, and fixation security during the clinical use of amniotic membrane grafts.

This analysis aligned all curves by behavior to reduce toe-region variability and improve comparability across specimens. Although this approach enabled fairer comparison of the monotone loading branch, it also normalized the strain span and may therefore have reduced sensitivity to absolute differences in extensibility. In addition, the modest sample size and the use of multiple correlated mechanical metrics limit the strength of statistical inference. For this reason, the present mechanical findings should be interpreted as exploratory and hypothesis-generating, with effect-size estimates providing a quantitative basis for the design of adequately powered confirmatory studies. Future work should increase the number of donors, refine thickness and area measurements, and incorporate complementary viscoelastic protocols, such as stress relaxation or cyclic preconditioning, to better characterize the time-dependent mechanical behavior of decellularized hAM.

## 5. Conclusions

This study demonstrates that the evaluated enzymatic decellularization methods based on trypsin and thermolysin preserve the extracellular matrix composition of the human amniotic membrane, particularly key proteins such as laminin and fibronectin, thereby supporting its potential use as a biological scaffold in tissue engineering.

However, both protocols induce differential effects on tissue structure and mechanical properties. Trypsin showed greater effectiveness in epithelial removal, representing a more efficient decellularization method, whereas thermolysin produced greater stromal alteration, compromising structural integrity to a greater extent.

From a biomechanical perspective, both treatments led to a reduction in mechanical performance compared to native tissue, consistent with a softening effect associated with the decellularization process. Nevertheless, these alterations were method-specific: thermolysin primarily reduced early- and mid-path stiffness, while trypsin had a greater impact on late-stage resistance and energy absorption capacity.

Taken together, these findings demonstrate that no single decellularization protocol is universally optimal; instead, method selection should be guided by achieving a balance between efficient cellular removal and preservation of the structural and functional properties of the tissue, depending on its intended application.

Finally, this study highlights the importance of a comprehensive evaluation of decellularization protocols through a multidimensional approach, incorporating histological, biochemical, and mechanical analyses, thereby contributing to the rational design of more effective and reproducible biomaterials for regenerative medicine.

## Figures and Tables

**Table 1:** Results of the integrity score for nhAM and dhAM.

Number of hAM *	Epithelium	Average Epithelium	Stroma	Average Stroma
1	1	0, 33	0	0, 00
	0		0	
	0		0	
2	0	0, 00	0	0, 00
	0		0	
	0		0	
3	3	3	0	0, 33
	3		1	
	3		0	
4	3	3	0	0
	3		0	
	3		0	
5	3	3	1	1
	3		1	
	3		1	
6	3	3	0	0, 67
	3		1	
	3		1	
7	3	3	1	1
	3		1	
	3		1	
8	3	2, 67	1	1
	3		1	
	2		1	
9	3	2, 67	1	1
	2		1	
10	3	3	1	0, 33
	3		0	
	3		0	

* Number of hAM. 1–2 no-decellularized hAM, 3–6 decellularized with trypsin, and 7–0 decellularized with thermolysin.

**Table 2:** Epithelial and stromal integrity scores in untreated nhAM and hAM decellularized with trypsin or thermolysin *.

Group	n	Epithelium Score, Median (Q1–Q3)	Stromal Score, Median (Q1–Q3)
nhAM (control)	2	0.17 (0.08–0.25)	0.00 (0.00–0.00)
dhAM with tripsin	4	3.00 (3.00–3.00)	0.50 (0.25–0.75)
dhAM with thermolysin	4	2.84 (2.67–3.00)	1.00 (0.83–1.00)

* Data are presented as median and interquartile range (Q1–Q3). Each value represents the mean integrity score obtained from three microscopic fields evaluated in each hAM sample. The hAM sample was considered the experimental unit.

**Table 3:** Quantitative image analysis of fibronectin and laminin α immunolabeling in nhAM and dhAM.

			Fibronectin			Laminin α	
hAM Sample	Group	Positive Area	Positive Area (%)	IOD	Positive Area	Positive Area (%)	IOD
1	nhAM	147,499.53	97.57	36,698,590.53	147,023.26	96.97	37,490,932.38
1	nhAM	125,787.00	100.00	31,362,138.00	147,463.96	97.26	37,603,309.43
1	nhAM	144,408.77	97.90	36,824,236.63	144,951.66	95.60	36,962,674.18
2	dhAM with trypsin	145,072.60	98.34	36,993,512.50	146,942.32	99.60	37,470,291.68
2	dhAM with trypsin	143,649.72	97.37	36,630,679.42	145,717.30	98.77	37,157,911.90
2	dhAM with trypsin	145,980.84	98.95	37,225,113.18	146,414.31	99.25	37,335,649.86
3	dhAM with thermolysin	144,628.21	98.03	36,880,194.45	147,022.19	99.66	37,490,658.43
3	dhAM with thermolysin	143,429.11	97.22	36,574,424.14	146,460.99	99.28	37,347,552.89
3	dhAM with thermolysin	124,457.00	98.94	31,736,739.00	147,179.23	99.76	37,530,704.05

**Table 4:** Group-wise mean ± SD (and n) for selected metrics.

Group of hAM	E_tan (ξ = 0.05) [MPa]	E_sec (ξ = 0.50) [MPa]	σ (ξ = 0.75) [MPa]	Work Density [MPa]
Untreated (without decellularizing)	2.63 ± 1.84 (n = 3)	1.91 ± 1.26 (n = 3)	0.243 ± 0.168 (n = 3)	0.0382 ± 0.0105 (n = 3)
Trypsin decellularization	2.59 ± 2.7 (n = 5)	1.57 ± 2.34 (n = 5)	0.0992 ± 0.0888 (n = 5)	0.0121 ± 0.0164 (n = 5)
Thermolysin descellularization	0.69 ± 0.662 (n = 5)	1.14 ± 1.07 (n = 5)	0.188 ± 0.144 (n = 5)	0.0179 ± 0.0178 (n = 5)

**Table 5:** Treatments Vs. control comparisons for key metrics.

	hAM + Trypsin vs. hAM Non-Decellularized				hAM + Thermolysin vs. hAM Non-Decellularized			
Metric	E_tan (ξ = 0.05)	E_sec (ξ = 0.50)	σ (ξ = 0.75)	Work density	E_tan (ξ = 0.05)	E_sec (ξ = 0.50)	σ (ξ = 0.75)	Work density
Δ (treat–control)	−0.03906	−0.3401	−0.1435	−0.02611	−1.942	−0.7717	−0.05481	−0.02032
*p*-value	0.967	0.966	0.160	0.073	0.070	0.165	0.646	0.139
Hedges’ g	−0.01387	−0.1447	−1.03	−1.541	−1.414	−0.5897	−0.3122	−1.121
g 95% CI low	−1.676	−6.791	−3.981	−11.77	−8.569	−3.300	−1.917	−5.732
g 95% CI high	1.195	0.7758	0.05946	−0.514	−0.09728	0.649	1.014	−0.135
Effect size	negligible	negligible	large	large	large	medium	small	large

**Table 6:** ε′UTS summary by group.

Group of hAM	ε′UTS Mean	eps_fail_sd
Non-decellularized	0.3053	0.07801
Decellularized with trypsin	0.2575	0.1952
Decellularized with thermolysin	0.1932	0.05646

## Data Availability

The original contributions presented in this study are included in the article. Further inquiries can be directed to the corresponding author.

## Abbreviations

The following abbreviations are used in this manuscript:

DAB	3, 3′-diaminobencidin
dhAM	Decellularize human amniotic membrane
DMEM	Dulbecco’s Modified Eagle Medium
ECM	Extracellular matrix
EDTA	Etilendiaminotetraacétic acid
E_sec	Engineering strain
E_tan	Tangent modulus
FPCA	Functional principal component analysis
H&E	Hematoxylin & Eosin
HUS	Hospital Universitario La Samaritana
hAM	Human amniotic membranes
MH	Meyer’s Hematoxylin
MPa	Mega pascals
nhAM	No decellularize human amniotic membrane
PBS	Phosphate-Buffered Saline
UTS	Ultimate tensile stress
